# Multimodal graph neural networks in healthcare: a review of fusion strategies across biomedical domains

**DOI:** 10.3389/frai.2025.1716706

**Published:** 2026-01-09

**Authors:** Maria Vaida, Ziyuan Huang

**Affiliations:** 1 Department of Analytics, Harrisburg University of Science and Technology, Harrisburg, PA, United States; 2 Department of Emergency Medicine, UMass Chan Medical School, Worcester, MA, United States

**Keywords:** graph neural networks, multimodal fusion, healthcare applications, biomedical data integration, attention mechanisms, drug discovery, cancer prognosis, neurological disorders

## Abstract

Graph Neural Networks (GNNs) have transformed multimodal healthcare data integration by capturing complex, non-Euclidean relationships across diverse sources such as electronic health records, medical imaging, genomic profiles, and clinical notes. This review synthesizes GNN applications in healthcare, highlighting their impact on clinical decision-making through multimodal integration, advanced fusion strategies, and attention mechanisms. Key applications include drug interaction and discovery, cancer detection and prognosis, clinical status prediction, infectious disease modeling, genomics, and the diagnosis of mental health and neurological disorders. Various GNN architectures demonstrate consistent applications in modeling both intra- and intermodal relationships. GNN architectures, such as Graph Convolutional Networks and Graph Attention Networks, are integrated with Convolutional Neural Networks (CNNs), transformer-based models, temporal encoders, and optimization algorithms to facilitate robust multimodal integration. Early, intermediate, late, and hybrid fusion strategies, enhanced by attention mechanisms like multi-head attention, enable dynamic prioritization of critical relationships, improving accuracy and interpretability. However, challenges remain, including data heterogeneity, computational demands, and the need for greater interpretability. Addressing these challenges presents opportunities to advance GNN adoption in medicine through scalable, transparent GNN models.

## 1 Introduction

Graphs serve as fundamental mathematical structures for representing and analyzing the complex relationships inherent in multimodal datasets. In the healthcare domain, nodes in a graph can represent medical entities such as patients, diseases, genes, proteins, medications, and healthcare providers, while edges capture the associations or interactions among them (Paul et al., 2024). Node and edge features may incorporate additional attributes, including patient demographic details, disease states, medical notes, or medication properties (Li et al., 2023a). Traditional machine learning and deep learning techniques, designed primarily for Euclidean data, often struggle to accommodate the non-Euclidean nature of relational medical data. GNNs address this limitation by extending deep neural networks to graph-structured data by aggregating and propagating information from neighboring nodes to learn high-order interactions through methods such as contrastive, generative, and explainable GNNs (Kumar et al., 2023b; Sefer, 2025b,a; Cetin and Sefer, 2025). This enables GNNs to generate graph-level representations that capture the structural and semantic complexity of medical data (Lee et al., 2024a; Kumar et al., 2023b). GNNs have proven effective in a wide range of healthcare applications, from disease diagnosis and comorbidity prediction to patient referral optimization and emotional intelligence modeling in clinical settings (Sangeetha et al., 2024; Pablo et al., 2024; Wang, 2022; Xu et al., 2024).

Healthcare data is inherently diverse and often available in multiple modalities, including structured data like EHRs, unstructured data like clinical notes, and complex forms like medical images (MRI, CT, PET, EEG, MEG), chemical, laboratory, temporal, and genomic data. Integrating and analyzing these heterogeneous data sources is crucial for a holistic understanding of disease and patient conditions. Multimodal learning, which aims to leverage complementary information from different modalities, is a logical tool for incorporating these disparate data sources (Waqas et al., 2024; Stahlschmidt et al., 2022; Teoh et al., 2024; Dumyn et al., 2024). GNNs are particularly well suited for multimodal healthcare applications, as they can model the intricate relationships within and between these diverse data streams and can be fused together with other deep learning or machine learning models (Dawn et al., 2024; Paul et al., 2024; Johnson et al., 2024).

This paper provides a review of the recent applications of GNNs in healthcare, with a specific focus on approaches that incorporate multimodal data. We structure the review by grouping applications into key themes: pharmacology, oncology, epidemiology, neuropsychiatry, clinical risk prediction, and genomics. By examining the methodologies, findings, and challenges within each area, this review aims to offer a comprehensive overview of the current landscape and potential future directions for GNNs in computational healthcare.

We defined the scope in advance to include primary studies that (1) apply a graph neural network to a biomedical or clinical task and (2) integrate at least two data modalities or combine graph learning with other encoders within an explicit fusion scheme. We searched PubMed, Google Scholar, and arXiv for studies published between January 2020 and August 2025 using combinations of graph-learning terms (e.g., GNN, GCN, GraphSAGE, GAT, heterogeneous graph), multimodality terms (e.g., multimodal, fusion), and health-domain terms (e.g., clinical, oncology, pharmacology, genomics). Titles and abstracts were screened against predefined inclusion and exclusion criteria, followed by full-text assessment. We included studies that reported the fusion strategy and described the architectural components used; single-modality GNNs, non-health domains, and papers lacking full text were excluded. The search identified 121 records, of which 85 studies met the eligibility criteria and were included in the review. Because reporting practices and evaluation metrics vary widely across domains, we used descriptive synthesis rather than quantitative meta-analysis. Complete search strings and eligibility details are provided in Supplementary Tables S1–S3.

## 2 Pharmacology

Pharmacology-focused multimodal GNN frameworks unify molecular, biological, and clinical signals under predominantly intermediate, attention-aware fusion, with early fusion used when EHR/image or graph features are concatenated prior to graph convolutions (Table 1). Heterogeneous graphs (drugs–targets–diseases–genes–adverse events), patient/population graphs, meta-path encoders with explainable decoders, and attention are common graph modeling approaches (Gao Y. et al., 2025; Huang et al., 2023; Zhou et al., 2024; Dawn et al., 2024). Drug-drug interaction models integrate drug–protein–disease multiplexes with multi-head attention, temporal or GNN/DNN pipelines, and graph transformers (Yu et al., 2023; Gan et al., 2023; Al-Rabeah and Lakizadeh, 2022; ChandraUmakantham et al., 2024; Wang G. et al., 2024; Xiong et al., 2023). Drug–target affinity prediction tasks fuse molecular graphs with knowledge-graph embeddings and attention modules (Yella et al., 2022; Zhang et al., 2023b; Xiang et al., 2025). Drug repurposing leverages knowledge-graph VAEs/GraphSAGE over drug databases to prioritize candidates, while adversarial designs extend to adverse events prediction and drug recommendations (Hsieh et al., 2020, 2021; Artiñano-Muñoz et al., 2024; Lin et al., 2023; Abdeddaiem et al., 2025). Time series and causal structure are explicit in models that learn temporal edges or motif-level constraints (e.g., CT-GNN/MDTCKGNN) and in prescription prediction with time-aware modules (T-LSTM) (Kalla et al., 2023; Liu et al., 2020). Vision-centric tasks (pill classification) add ConvNet/RPN with graph topology learning. Protein localization alteration and colonization-risk models adapt GraphSAGE/GCN/GAT to dynamic clinical graphs (Nguyen et al., 2023; Wang R. H. et al., 2023; Gouareb et al., 2023).

**Table 1:** Graph-based models across pharmacology-related tasks.

**Task**	**Model**	**Fusion**	**Dataset**	**Layers**	**AUC**	**F1**	**Accuracy**
Adverse drug event (Gao Y. et al., 2025)	PreciseADR	Early	FAERS adverse drug events, demographics, diseases, drugs	HGNN	0.54–0.84	NR	NR
Adverse drug event (Kalla et al., 2023)	MDTCKGNN	Intermediate	ade_corpus_v2; PHEE	HGNN with attention	NR	NR	NR
Adverse drug event (Zhou et al., 2024)	Patient-centric GNN	Early	Australian CBHS	GraphSAGE with attention	0.88–0.96	0.53–0.90	0.89–0.94
Adverse drug event (Huang et al., 2023)	HHAN-DSI	Intermediate	SIDER, OFFSIDE, GO	HGNN + TransE	0.84–0.94	NR	NR
Adverse drug event (Dawn et al., 2024)	MI-GNN	Intermediate	Decagon DDI	HGNN	0.81–0.95	NR	NR
Drug Repurposing (Artiñano-Muñoz et al., 2024)	DRAGON	Intermediate	DISNET	GraphSAGE	0.91–0.95	NR	NR
Drug repurposing (Hsieh et al., 2020)	COVID-19 KG	Intermediate	Literature, CTD	VGAE, GraphSAGE	0.78–0.90	NR	NR
Drug repurposing (Hsieh et al., 2021)	SARS-CoV2 KG	Intermediate	Literature, CTD	VGAE, GraphSAGE	0.77–0.90	NR	NR
Drug repurposing (Abdeddaiem et al., 2025)	AGMR	Early	MIMIC-III	GNN, GAN	NR	0.85–0.88	0.86–0.88
Drug repurposing (Lin et al., 2023)	AD Drug Repurposing	Early	STRING, GO, CTD	GraphSAGE	0.84–0.99	NR	NR
Drug–Drug Interaction (Gan et al., 2023)	DMFDDI	Intermediate	Zhang, ChCh-Mine, DeepDDI	Temporal HGNN	0.95–0.99	0.93–0.97	NR
Drug–drug interaction (Yu et al., 2023)	ACDGNN	Intermediate	Gene/disease/pathway KG	HGNN with attention	0.71–0.99	0.67–0.94	0.67–0.97
Drug–drug interaction (Al-Rabeah and Lakizadeh, 2022)	GNN-DDI	Intermediate	DrugBank, KEGG	HGNN	0.99–1.00	0.41–0.86	0.67–0.92
Drug–drug interaction (ChandraUmakantham et al., 2024)	DeepSide	Intermediate	TwoSides; DrugBank	GraphSAGE with attention	NR	0.83–0.99	0.77–0.99
Drug–drug interaction (Wang G. et al., 2024)	MMDDI-MGPFF	Intermediate	DrugBank	GINConv with attention	NR	0.96	0.88
Drug–drug interaction (Xiong et al., 2023)	MRCGNN	Intermediate	Deng, Ryu datasets	TrimNet + GNN	NR	0.78–0.89	0.89–0.90
Drug–target prediction (Zhang et al., 2023b)	DrugAI	Intermediate	DrugBank	AttentiveFP, LINE, DeepWalk, node2vec, SDNE	0.88–0.97	0.87–0.89	0.85–0.93
Drug–target prediction (Xiang et al., 2025)	ExplainMIX	Intermediate	CCLE, GDSC, PubChem	RGCN	0.00–1.0	0.73–0.97	NR
Drug–target prediction (Yella et al., 2022)	GraMDTA	Intermediate	DrugBank, RepoDB, DisGeNET	CNN, GraphSAGE with attention	0.88–0.92	0.69–0.80	NR
Pill classification (Nguyen et al., 2023)	PGPNet	Intermediate	User-captured pill images	ConvNet, RPN, GTN	NR	NR	0.70–0.90
Prescription prediction (Liu et al., 2020)	RGNN	Intermediate	MIMIC-III	T-LSTM, Temporal GNN	0.82–0.84	NR	NR
Protein localization alteration (Wang R. H. et al., 2023)	PLA-GNN	Early	GEO	GraphSAGE	NR	NR	0.410–0.41

NR, Not reported by the original study.

GraphSAGE/GCN/GAT/RGCN provide the backbone of drug-related multimodal GNN approaches, with attention (often multi-head) capturing neighbor weighting and modality selection. VGAE/GAN variants aid representation learning and robustness (Yu et al., 2023; Wang G. et al., 2024; Xiang et al., 2025; Abdeddaiem et al., 2025). Datasets span FAERS, SIDER/OFFSIDES/TWOSIDES, DrugBank, KEGG, STRING, CCLE/GDSC, KIBA/DAVIS, RepoDB, and MIMIC-III/MIMIC-IV, enabling cross-domain evaluation from molecules to bedside (Gao Y. et al., 2025; Dawn et al., 2024; Al-Rabeah and Lakizadeh, 2022; Yella et al., 2022; Zhang et al., 2023b; Liu et al., 2020). Recent surveys have argued that multimodal, knowledge-graph-aware, and temporally grounded GNNs tend to improve property prediction, DDI/ADE surveillance, repurposing, and recommendation while enhancing mechanistic insight and scalability (Paul et al., 2024; Tabatabaei et al., 2025; Yao et al., 2024; Wang Y. et al., 2024; Li et al., 2023a).

## 3 Oncology

Oncology-focused multimodal graph frameworks fuse histopathology, radiology, omics, and clinical covariates to support diagnosis, risk stratification, and treatment planning tasks (Table 2). Most systems pair modality-specific encoders, such as CNNs/ViTs or radiomics for images, text encoders for reports, and pathway/interaction graphs for omics, with graph layers under intermediate fusion, frequently using attention for weighting (Kulandaivelu et al., 2024; Kim et al., 2023; Alzoubi et al., 2024; Pratap Joshi et al., 2025; Yan et al., 2024; Gowri et al., 2024). Population graphs connect patients via various similarity measures in imaging and clinical embeddings (head and neck, ovarian cancers), while pathways and knowledge graphs encode gene–gene or entity relations for subtype and survival modeling (Peng et al., 2024; Ghantasala et al., 2024; Li et al., 2023b). Lesser used, late fusion is applied when independently learned patient–gene bipartite embeddings are aligned for survival (MGNN) (Gao et al., 2022), whereas early fusion concatenates raw/image features before graph reasoning in lung and federated liver cancer models (Li et al., 2023b; Moharana et al., 2025). Beyond core oncology tasks, misinformation detection integrates text encoders with R-GCN over medical knowledge graphs under early fusion (Cui et al., 2020). These architectures standardize heterogeneous inputs, learn structure-aware patient and pathway representations, and improve generalization via similarity graphs and attention-based aggregation across modalities and fusion types (Li et al., 2023a; Paul et al., 2024; Waqas et al., 2024).

**Table 2:** Graph-based models across oncology-related tasks.

**Task**	**Model**	**Fusion**	**Dataset**	**Layers**	**AUC**	**F1**	**Accuracy**
Breast cancer (Gao et al., 2022)	MGNN	Late	2,500 breast cancer patients with gene expression, CNA, and clinical data	Temporal GNN on bipartite graphs, CCA fusion	0.98	NR	0.95
Breast cancer (Kulandaivelu et al., 2024)	ABCD-HAHGNN-MI	Intermediate	DDSM and CBIS-DDSM	DNPGF, QOLCT, GLCM, SCL, CAL, GNN with attention	0.94–0.97	0.95–0.97	0.97–0.98
Breast cancer (Kim et al., 2023)	HetMed	Intermediate	Duke-Breast and CMMD	ResNet, CNN, GCN with attention	NR	0.70–0.86	NR
Glioma (Alzoubi et al., 2024)	PathoFusion	Intermediate	WSI pathology images	CNN, GCN with attention	NR	NR	0.83–0.85
Glioma (Pratap Joshi et al., 2025)	VSA-GCNN	Intermediate	BraTS 2019, 2020, 2021	AlexNet, VSA, GCN with attention	NR	0.97–0.98	0.92–1.00
Lung cancer (Li et al., 2023b)	Lung adenocarcinoma multiclassification model	Early	Zhongshan Hospital; Shanghai Public Health Clinical Center	CNN, GIN, GNN with attention	0.92–0.95	NR	0.87–0.95
Head and neck cancer (Peng et al., 2024)	MLF-GNN	Intermediate	TCIA	GraphSAGE, GNN with attention	NR	NR	0.85–0.94
Liver cancer (Moharana et al., 2025)	FML-LDP	Early	Clinical, demographic, genetic, and imaging data	CNN, GNN with attention, federated meta-learning	NR	0.85–0.93	0.92–0.97
Ovarian cancer (Ghantasala et al., 2024)	Temporal Analysis + GNN	intermediate	OCD and NCI SEER	RNN, GNN with attention	0.60–0.82	0.56–0.78	0.56–0.79
Skin cancer (Yan et al., 2024)	MSF-CNN	Intermediate	ISIC dataset	CNN, GNN with attention	0.66–0.76	0.55–0.63	0.77–0.82
Multi-cancer detection (Gowri et al. 2024)	Vision transformers + GNNs + LayoutLM	Intermediate	IQ-OTH/NCCD Lung Cancer Dataset; PLCO Lung Dataset	ViTs, GNN, LayoutLM	NR	NR	NR
Oncology Misinformation Detection (Cui et al., 2020)	DETERRENT	Early	KnowLife, Healthline, ScienceDaily, NIH, MNT, Mayo Clinic, Cleveland Clinic, WebMD	BiGRU, RGCN with attention	0.54–0.83	0.28–0.67	0.44–0.70

NR, Not reported by the original study.

## 4 Neuropsychiatry

Multimodal GNN frameworks extended to neurological domains have been applied to conditions such as Alzheimer's disease, Parkinson's disease, depression, autism spectrum disorder, Schizophrenia, and even emotion recognition and sentiment analysis by integrating diverse linguistic, genomic, behavioral, imaging, and physiological data (Teoh et al., 2024; Zhang et al., 2023a; Xu et al., 2024; Sangeetha et al., 2024; Khemani et al., 2024).

Neuropsychiatry multimodal GNN pipelines unify imaging (fMRI/sMRI/DTI/PET), electrophysiology (EEG), speech/text, and omics within subject or population-level graphs (Table 3). A common approach in Alzheimer's disease prediction integrates imaging-driven fusion with cross-attention Transformers (CsAGP, GCNCS), dual hypergraphs (DHFWLSL), multiplex subject graphs (HetMed), and hypergraph attention fusion (HCNN-MAFN) (Tang C. et al., 2023; Luo et al., 2024; Kim et al., 2023; Kumar et al., 2023a; Lee et al., 2024b). Parkinson's studies pair connectomic encoders with omics via attention (JOIN-GCLA) and patient-similarity graphs (AdaMedGraph) (Chan et al., 2022; Lian et al., 2023). Autism Spectrum Disorder models treat rs-fMRI as signals on DTI graphs (M-GCN) to intermediate spatio-temporal/demographic fusion (IFC-GNN) and VAE-aligned Transformer/Graph-U-Net encoders (MM-GTUNets) (Dsouza et al., 2021; Wang X. et al., 2024; Cai et al., 2025). For Major Depressive Disorder, interview-centric systems employ heterogeneous attention over audio–video–text (AVS-GNN, DSE-HGAT), while imaging/population approaches (LGMF-GNN, FC-HGNN, Ensemble GNN) couple local ROI graphs to global subject graphs (Li et al., 2025, 2024; Liu et al., 2024; Gu et al., 2025; Venkatapathy et al., 2023; Lee et al., 2024a). Schizophrenia pipelines tend to model EEG channel-graphs and dual-branch DTI attention networks integrating FA/FN features (Jiang et al., 2023; Gao et al., 2025). Attention weights filter population graphs based on their similarity, and learn multi-scale spatial–temporal patterns by combining CNN/Transformer encoders with GNN message passing inside the fusion stack.

**Table 3:** Graph-based models across neuro/psychiatric tasks.

**Task**	**Model**	**Fusion**	**Dataset**	**Layers**	**AUC**	**F1**	**Accuracy**
Alzheimer's (Cai et al., 2023)	AD-GNN	Intermediate	Augmented Pitt Cookie-Theft dataset	BERT, GraphSAGE, BiLSTM, GGNN	NR	NR	0.77–0.85
Alzheimer's (Wang Z. et al., 2024)	Knowledge-infused MM-GNN	Intermediate	OASIS; ADNI-D	LLMs, GNN	0.46–0.67	0.46–0.68	0.55–0.82
Alzheimer's (Tang C. et al., 2023)	CsAGP	Intermediate	ADNI (ADNI1/GO and ADNI2)	CNN, Vision Transformers, GNN with attention	0.99–1.00	NR	0.94–0.99
Alzheimer's (Luo et al., 2024)	DHFWLSL	Intermediate	ADNI (ADNI1/GO and ADNI2)	Dual HGNN with Laplacian regularization	NR	0.42–0.93	0.51–0.94
Alzheimer's (Kim et al., 2023)	HetMed	Intermediate	ADNI	HGNN, CNN (ResNet), GNN with attention	NR	0.70–0.86	NR
Alzheimer's (Kumar et al., 2023a)	HCNN-MAFN	Intermediate	ADNI	HGNN with attention	0.98–0.99	0.94–0.96	0.94–0.96
Alzheimer's (Lee et al., 2024b)	GCNCS	Intermediate	ADNI and DAUH	CNN, GNN	0.92–0.97	0.90–0.99	0.88–0.94
Alzheimer's (Tripathy et al., 2025)	GNNRAI	Intermediate	ROSMAP, MSBB, Mayo	GNN with attention	0.95–1.00	0.95–1.00	0.76–1.00
Parkinson's (Lian et al., 2023)	AdaMedGraph	Early	PPMI and PDBP	GNN	0.65–0.76	NR	NR
Parkinson's (Chan et al., 2022)	JOIN-GCLA	Intermediate	PPMI	GNN with attention	NR	NR	0.90–1.00
Neurodegenerative (Vijay Anand et al., 2024)	IMNMAGN	Intermediate	BioGPS and BrainLat	ICA, Correlation Analysis, TFA, Beamforming, CNN, GNN with attention	0.95–0.97	NR	0.91–0.97
Autism (ASD) (Dsouza et al., 2021)	M-GCN	Early	HCP and KKI	GCN	NR	NR	NR
Autism (ASD) (Wang X. et al., 2024)	IFC-GNN	Intermediate	ABIDE I	Temporal GNN	NR	NR	0.64–0.81
Autism (ASD) (Cai et al., 2025)	MM-GTUNets	Intermediate	ABIDE I and ADHD000	VAE CNN, RL Q-Learning, GNN with attention	0.88–0.91	NR	0.82–0.83
Major depressive disorder (Li et al., 2025)	AVS-GNN	Intermediate	DAIC-WOZ and DVlog	LSTM, GNN, MLP	NR	0.74–0.88	0.75–0.86
Major depressive disorder (Xing et al., 2024)	EMO-GCN	Intermediate	MODMA	GraphSAGE, GNN with attention	NR	0.89–0.96	0.90–0.97
Major depressive disorder (Liu et al., 2024)	LGMF-GNN	Intermediate	SRPBS and REST-meta-MDD	BiGRU, Snowball GNN	0.73–0.81	0.65–0.91	0.70–0.79
Major depressive disorder (Venkatapathy et al., 2023)	Ensemble GNN	Intermediate	REST-meta-MDD	GNN with attention and GraphSAGE	0.71–0.77	NR	0.70–0.72
Major depressive disorder (Gu et al., 2025)	FC-HGNN	Intermediate	ABIDE and REST-meta-MDD	GNN with attention	0.95–1.00	0.93–1.00	0.92–1.00
Major depressive disorder (Lee et al., 2024a)	Spectral GNN	Early/Late	REST-meta-MDD	Spectral GNNs	0.66–0.74	NR	0.67–0.73
Major depressive disorder (Li et al., 2024)	DSE-HGAT	Intermediate	DAIC-WOZ	BiLSTM, GNN with attention	NR	0.79	NR
Schizophrenia (Jiang et al., 2023)	Multimodal GNN for EEG	Early/ Intermediate	Chengdu, Hangzhou, Moscow datasets	GNN	0.70–0.85	NR	0.70–0.88
Schizophrenia (Gao et al., 2025)	GNN and Multimodal DTI	Intermediate	7 sites across China	GNN with attention	NR	0.74–0.76	0.71–0.74

NR, Not reported by the original study.

## 5 Epidemiology

Recent epidemic-forecasting and COVID-19 outcome models fuse temporal sequence encoders with structure-aware GNNs (Table 4). For population-level spread, architectures stack temporal CNN/DNN modules with attention-based GNN layers to capture local and global transmission patterns (MSGNN, EpiGNN) and augment signals with LLM-derived social media features or dual topologies to improve influenza forecasts (MGLEP, Dual-Topo-STGCN) (Qiu et al., 2024; Xie et al., 2023; Tran et al., 2024; Luo et al., 2025). Within hospitals, contact graphs linking patients and healthcare workers use GraphSAGE and attention to model hospital-acquired infection transmission (Gouareb et al., 2023). For COVID-19 prognosis, multimodal pipelines use attention to fuse CT-derived features with KNN population graphs (Keicher et al., 2023), while edge-flexible GCNN frameworks integrate imaging, tabular, and temporal signals (CNN/LSTM and population GNN) to allow post-training edge adaptability (Tariq et al., 2023, 2025). These models emphasize spatiotemporal message passing, attention for weighting neighbors and signals, and adaptable graph construction to handle dynamic data.

**Table 4:** Graph-based models for epidemic forecasting and outcomes.

**Task**	**Model**	**Fusion**	**Dataset**	**Layers**	**AUC**	**F1**	**Accuracy**
Epidemic forecasting (Qiu et al., 2024)	MSGNN	Intermediate	JHU CSSE	Temporal CNN, GNN with attention	NR	NR	NR
Epidemic forecasting (Xie et al., 2023)	EpiGNN	Intermediate	COVID Japan-Prefectures, ILINet, ILI, JHU-CSSE, Spain-COVID	CNN, AutoregressiveDNN, and GNN with attention	NR	NR	NR
Epidemic forecasting (Tran et al., 2024)	MGLEP	Intermediate	JHU CSSE, OxCGRT, COVID-19 Twitter chatter	BertTweet, RNN, GNN with attention	NR	NR	NR
Epidemic forecasting (Luo et al., 2025)	Dual-Topo-STGCN	Intermediate	CDC ILI surveillance	RNN, GNN	NR	NR	NR
HAI transmission (Gouareb et al., 2023)	MDRE-TransGraph	Early	MIMIC-III	GNN with attention, GraphSAGE	0.89–0.96	NR	0.84–0.97
COVID-19 outcomes (Keicher et al., 2023)	Multimodal GAT	Intermediate	iCTCF and KRI	U-Net and KNN-based, GNN with attention	0.57–0.77	0.18–0.78	0.73–0.74
COVID-19 outcomes (Tariq et al., 2023)	GCNN for clinical event prediction	Intermediate	COVID-19 Emory University Hospital (EUH)	CNN, GNN, LSTM	0.50–0.91	NR	NR
COVID-19 outcomes (Tariq et al., 2025)	Adaptable GCNN for clinical event prediction	Intermediate	COVID-19 Emory University Hospital (EUH)	DenseNet-121, GraphSAGE, LSTM	0.58–0.92	NR	NR

NR, Not reported by the original study.

## 6 Clinical

EHR-based multimodal graph frameworks aim to support clinical prediction and treatment planning through merging diverse medical data modalities (Li et al., 2022; Xu et al., 2024). When combined with knowledge graphs, these models offer flexibility in terms of both inputs and prediction tasks (Nye, 2023; Rajabi and Kafaie, 2022). Most models integrate structured EHR (diagnoses, procedures, meds, labs, vitals) with at least one unstructured or high-dimensional stream, be it clinical notes, medical images (CXR, fundus), genomics, or wearable/sensor data, often via CNNs for imaging, TF-IDF/BioBERT for text, and temporal trajectory layers for labs/vitals (AL-Sabri et al., 2024; Tang S. et al., 2023; Zedadra et al., 2025; Pablo et al., 2024; Wang et al., 2025). The graph connectivity tends to be modeled as patient–patient similarity graphs, knowledge graphs linking encounters to conditions, and heterogeneous graphs (e.g., sensor and metapath views) (Table 5). Dynamic network edges implemented in conjunction with learned message-passing connectivity from static KGs allow graphs to adapt to new information without the need for retraining (Liu et al., 2021; Valls et al., 2023; Gao et al., 2024; Wang et al., 2025; Christos Maroudis et al., 2025).

**Table 5:** Graph-based models for clinical prediction, pathways, and hospital operations.

**Task**	**Model**	**Fusion**	**Dataset**	**Layers**	**AUC**	**F1**	**Accuracy**
Tuberculosis (D'Souza et al., 2023)	MaxCorr-MGNN	Intermediate	Tuberculosis Data Exploration Portal	Hirschfeld–Gebelein–Rényi maximal correlation and GNN	0.77–0.78	NR	NR
Care pathway prediction (Liu et al., 2021)	Multitask Healthcare Management System	Intermediate	600,000 multimodal samples (structured, text, images)	CNN (ResNet), GNN, Word2Vec, RNN	NR	NR	NR
Clinical risk prediction (AL-Sabri et al., 2024)	M3GNAS	Intermediate	MIMIC-III	BiGRU, BioBERT, GNN with attention	0.70–0.91	NR	NR
Hospital readmission (Tang S. et al., 2023)	MM-STGNN	Intermediate	MIMIC-IV; 9,958 admissions/44,084 radiographs/9,162 patients	GraphSAGE, RNN, GNN	0.58–0.91	NR	NR
Federated diagnosis (Begum, 2024)	FH-MMA	Intermediate	MIMIC-III	CNN, Transformers, and GNN with attention	NR	NR	0.93–0.95
Multitask longitudinal modeling (Boschi et al., 2024)	funGCN	Intermediate	SHARE and synthetic dataset	GNN	NR	NR	0.58–0.93
Clinical triage (Valls et al., 2023)	Masked-Connectivity Triage GNN	Intermediate	Synthea	GNN, KG, Temporal GNN	NR	NR	0.40–0.85
Comorbidity prediction (Pablo et al., 2024)	Multitask Comorbidity GCN	Intermediate	Imaging + genomics + clinical notes	CNN, BERT, GNN	0.96	0.93	0.95
Sleep apnea diagnosis (Wang et al., 2025)	HeteroGCFNet	Intermediate	OSAHS	BiLSTM, GNN with attention	NR	0.80–0.84	0.84–0.88
Sepsis trajectory modeling (Ghanvatkar and Rajan, 2023)	Dynamic Clinician-in-the-Loop GNN	Intermediate	MIMIC-IV	GNN, Temporal HGNN with attention	0.74	0.36	0.87
Ophthalmology auxiliary diagnosis (Gao et al., 2024)	CGAT-ADM	Intermediate	Ophthalmic EMRs (Beijing Tongren Hospital)	BERT, metapath2vec, GNN with attention	NR	NR	NR
Diabetic retinopathy (Zedadra et al., 2025)	DRdiag	Intermediate	APTOS 2019; MESSIDOR0	CNN, GNN	NR	0.96	0.96–0.98
Heart disease (Boll et al., 2025)	Patient-KNN Graph	Early	MIMIC-III	GraphSAGE, KNN, Graph Transformers, GNN with attention	0.75–0.79	0.47–0.53	0.70–0.80
ICU albumin prediction (Zhang et al., 2023)	DyG-HAP	Intermediate	ANIC	Disentangled dynamic graph with attention	NR	NR	NR
ICU length of stay (Christos Maroudis et al., 2025)	Fairness-Aware Dynamic ST-GNN	Intermediate	MIMIC-IV	LSTM, GNN with attention	0.82–0.91	NR	NR

NR, Not reported by the original study.

In terms of multimodal fusion strategies, the majority of models start with modality-specific encoders (CNNs for images, BiGRU/LSTM/Transformers for sequences/text), which are then integrated into GNN backbones (GraphSAGE, GNN/GAT, heterogeneous GNN), with attention used both for cross-modal weighting and within graph layers (AL-Sabri et al., 2024; Tang S. et al., 2023; Begum, 2024; Boschi et al., 2024; Ghanvatkar and Rajan, 2023). Temporal structure can be modeled at the node level (RNN/Transformer encoders per patient), edge level (temporal embeddings that define adaptive edges), and graph level (dynamic GNNs that rebuild neighborhoods by top-k similarity each step). Disentangled dynamic attention separates invariant vs. shifting patterns and fairness-aware designs (Tang S. et al., 2023; Zhang et al., 2023; Christos Maroudis et al., 2025).

MIMIC-III and MIMIC-IV are two of the most used datasets for mortality and length-of-stay prediction, as well as readmission, sepsis trajectory modeling, and heart-disease graphs, integrated with similarity-based measures, temporal encoders, dynamic graph update strategies, and privacy-preserving architectures (AL-Sabri et al., 2024; Tang S. et al., 2023; Ghanvatkar and Rajan, 2023; Christos Maroudis et al., 2025; Begum, 2024). Imaging-heavy models join population graphs with CNN/radiomics for tasks such as ophthalmology and DR screening (APTOS, MESSIDOR) (Gao et al., 2024; Zedadra et al., 2025), while sensor-centric pipelines exploit heterogeneous sensor-and-knowledge graphs (Wang et al., 2025). SHARE, Synthea, and ANIC datasets support multitask longitudinal modeling, ER triage, and out-of-distribution ICU biomarker forecasting (Boschi et al., 2024; Valls et al., 2023; Zhang et al., 2023).

By uniting EHR, imaging, genomic, temporal, and sensor-derived information within attention-based graph representations, diagnostics and prognostic models capture both the relational and temporal complexities inherent in patient care (Oss Boll et al., 2024). Their reliance on attention-based fusion and invariant pattern learning reflects a shift toward systems capable of modeling data heterogeneity and distribution shifts, resulting in scalable and generalizable clinical decision-support systems.

## 7 Genomics

Across lncRNA–miRNA interaction prediction, GNN models implement sequence-aware fusion with attention, built over heterogeneous similarity graphs (Table 6). Modalities and features typically combine primary sequence (k-mers), similarity networks (sequence/functional/disease), and structural or physicochemical descriptors into unified node–edge representations (Wang Z. et al., 2023; Wang et al., 2022; Wang and Chen, 2023; Zhang et al., 2022). Sequence embeddings are often initialized via unsupervized objectives (e.g., k-mer Doc2Vec) before graph learning, then refined with inductive backbones such as GraphSAGE and attention layers to weight informative neighbors (Wang Z. et al., 2023; Zhang et al., 2022). Heterogeneous/bipartite graphs integrate lncRNA–miRNA and miRNA–disease with similarity measures, structured probabilistic layers, or multi-channel attention (Wang et al., 2022; Wang and Chen, 2023). Datasets such as LncACTdb, LNCipedia, miRBased, ncRNASNP, and HMDD are integrated into pretrained sequence embeddings, heterogeneous similarity graphs, and attention-based GNNs to improve link prediction fidelity and mechanistic interpretability of gene expression.

**Table 6:** Graph-based models for ncRNA–miRNA interaction prediction.

**Task**	**Model**	**Fusion**	**Dataset**	**Layers**	**AUC**	**F1**	**Accuracy**
lncRNA–miRNA interaction (Wang Z. et al., 2023)	SPGNN	Intermediate	LncACTdb 3.0; LNCipedia; miRBase	k-mer Doc2Vec, GraphSAGE, GNN with attention	0.84	0.75–0.76	NR
lncRNA–miRNA interaction (Wang et al., 2022)	GCNCRF	Intermediate	lncRNASNP2; LncACTdb 3.0; LNCipedia; miRBase	Conditional Random Fields, GNN with attention	0.88–0.95	0.13–0.14	0.97–0.98
lncRNA–miRNA interaction (Wang and Chen, 2023)	MAGCN	Intermediate	ncRNASNP v2.0; HMDD v3.0	CNN, GNN with attention	0.90	0.50–0.51	0.94
ncRNA–miRNA interaction (Zhang et al., 2022)	ncRNAInter	Intermediate	lncRNASNP2; miRBase v22.1; GENCODE v38	GraphSAGE with neighbor sampling	0.97–0.99	0.93–0.96	0.93–0.96

NR, Not reported by the original study.

## 8 Discussion

**Figure 1:**
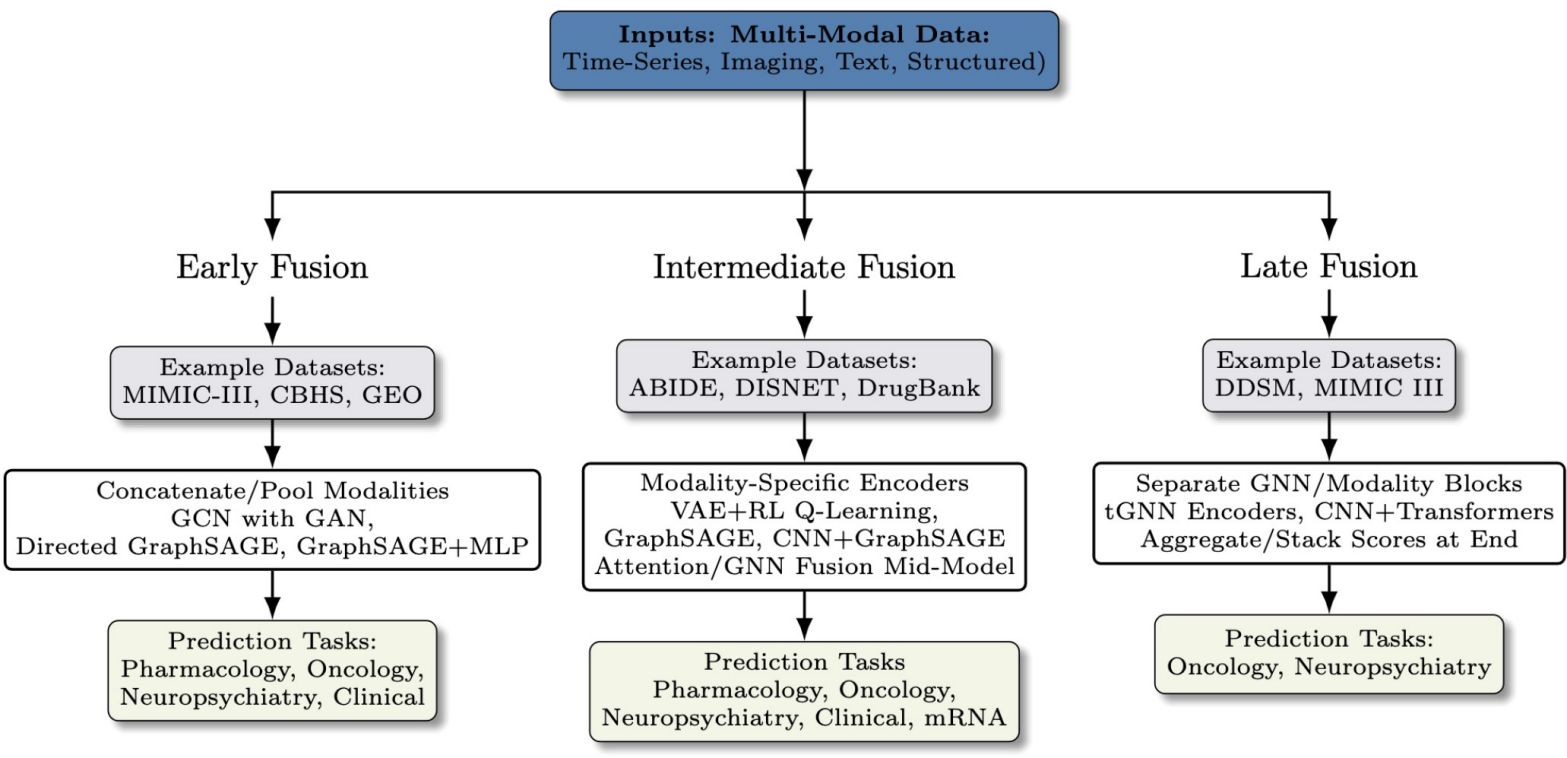
Conceptual workflow of multimodal fusion strategies. Early, intermediate, and late fusion integrate heterogeneous inputs for downstream prediction tasks. In early fusion, modalities are concatenated or pooled up front and passed to a unified encoder. In intermediate fusion, each modality is first processed by a modality-specific encoder, and features are combined mid-model via attention/GNN layers. In late fusion, separate modality/GNN branches are trained, and their scores are combined only at the decision stage. Prediction layers are dominated by fully connected layers, multiple-layer perceptrons, or machine learning classifiers.

Healthcare data is inherently multimodal, and integrating information from different sources can provide a more comprehensive view of a patient's health status or disease characteristics. Graph Neural Networks facilitate this by providing a framework to model relationships between and within each modality. The strengths of GNNs lie in their integration with other deep learning models by taking advantage of advanced fusion strategies, particularly those employing attention mechanisms. GNN integrations with CNNs, RNNs, autoencoders, language transformers, machine learning classification or regression models, and optimization algorithms facilitate multimodal data preprocessing and merging, as illustrated in the workflow of fusion types in Figure 1.

**Figure 2:**
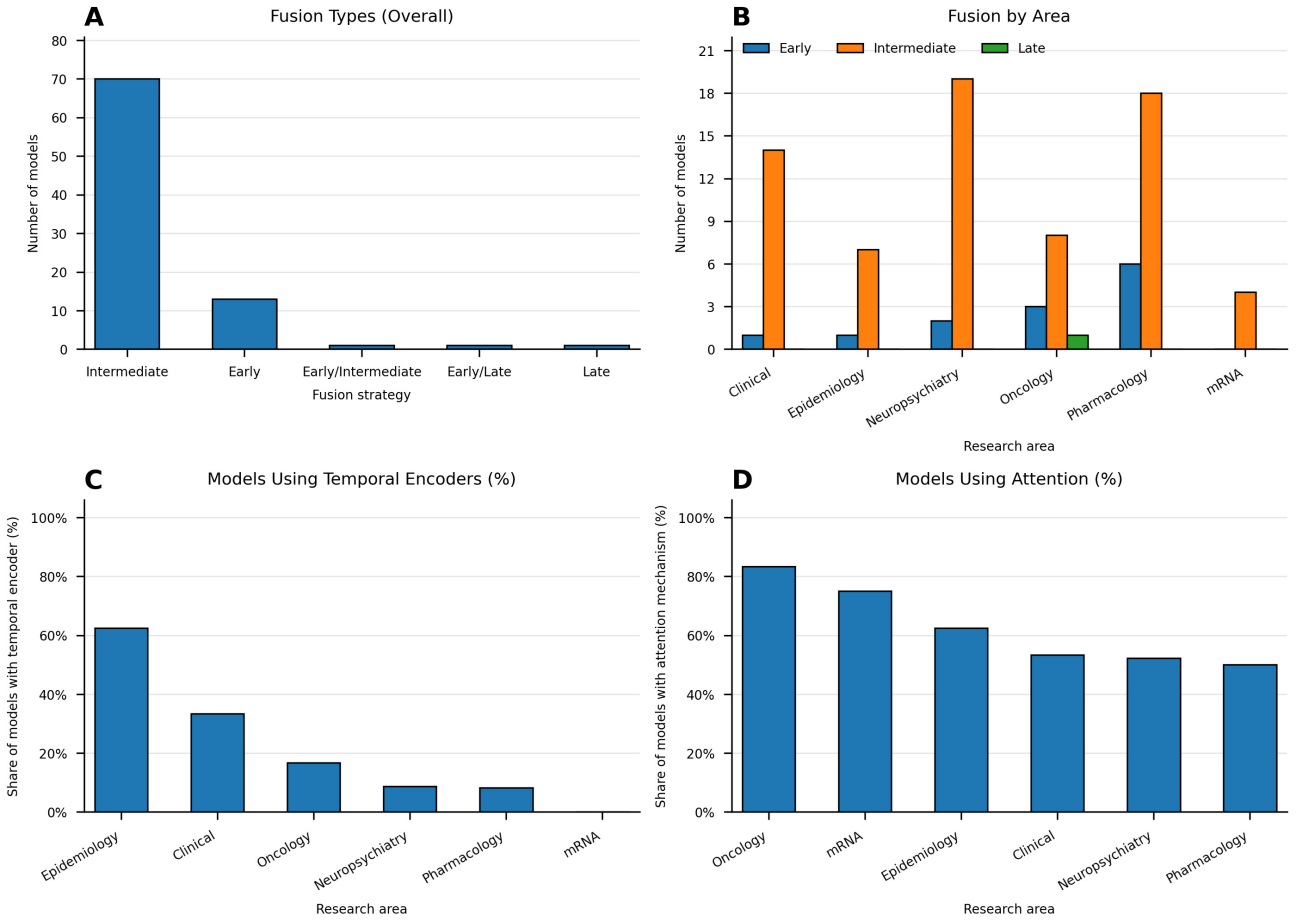
Multimodal fusion strategies and encoder usage across research areas. **(A)** Overall distribution of fusion strategies across all models. **(B)** Fusion distribution by area. **(C)** Share of models that include a temporal encoder by area. **(D)** Share of models that include an attention mechanism by area.

**Figure 3:**
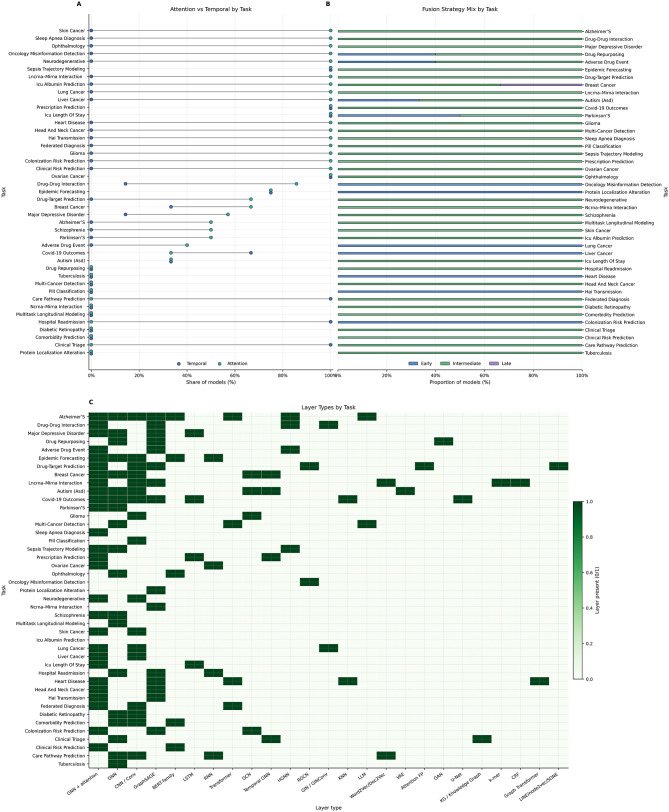
Architectural patterns across tasks. **(A)** Gap chart comparing the share of models using attention versus temporal encoders for the top tasks. **(B)** Normalized (100%) stacked bars showing the fusion strategy mix. Values are the proportion of models per task that use each fusion scheme. **(C)** Heatmap of layer types extracted from model descriptions.

Across research areas and prediction tasks, intermediate fusion is the prevailing design (Figures 2A, B, 3B). In epidemic forecasting, temporal encoders fuse data via attention-based graph layers to capture local and global spread (Qiu et al., 2024; Xie et al., 2023; Tran et al., 2024; Luo et al., 2025). Hospital-acquired infection models combine contact graphs with attention inside the graph pipeline (Gouareb et al., 2023). COVID-19 outcome prediction uses intermediate fusion that joins CT features with population graphs with adaptable edges (Keicher et al., 2023; Tariq et al., 2023, 2025). Clinical prediction and operations also favor intermediate fusion, where modality-specific encoders precede GraphSAGE, GCN, GAT, or heterogeneous GNN layers (AL-Sabri et al., 2024; Tang S. et al., 2023; Begum, 2024; Boschi et al., 2024; Valls et al., 2023; Zhang et al., 2023; Christos Maroudis et al., 2025). Oncology mostly follows the same pattern, with late fusion used when independent embeddings are aligned after training and early fusion used when features are concatenated before graph reasoning (Gao et al., 2022; Li et al., 2023b; Moharana et al., 2025; Alzoubi et al., 2024; Pratap Joshi et al., 2025; Peng et al., 2024; Yan et al., 2024). Gene expression studies implement sequence-aware intermediate fusion that mixes pretrained sequence embeddings with similarity graphs and attention (Wang Z. et al., 2023; Wang et al., 2022; Wang and Chen, 2023; Zhang et al., 2022).

Across the 85 studies reviewed, intermediate fusion accounts for 81% of models (*n* = 69), with the highest use in neuropsychiatry (83%) and pharmacology (74%), and attention layers are present in over 60% of systems. Early fusion constitutes 15% (*n* = 13), largely in oncology for raw feature concatenation. Late fusion appears in 1% (*n* = 1) for embedding alignment in genomics and hybrid fusion in 2% (*n* = 2), both in neuropsychiatry. Intermediate fusion is associated with the strongest outcomes, with top models reaching mean AUC values near 0.95 and accuracies near 0.92 (Table 7). Early fusion supports simpler feature integration with broader performance ranges (AUC 0.84–0.99), while late fusion suits alignment-driven tasks such as MGNN, where modality-specific embeddings are correlated only after independent training (AUC 0.98). Intermediate fusion consistently yields the most discriminative models, including Alzheimer's systems achieving AUC values up to 1.00, consistent with prior analyses of multimodal GNNs (Paul et al., 2024; Li et al., 2023a).

**Table 7:** Summary comparison of top-performing multimodal GNN models across biomedical domains, selected based on highest AUC, accuracy, and F1 scores, highlighting architectures, datasets, fusion types, and performance outcomes to identify effective strategies.

**Domain**	**Task**	**Model**	**Fusion**	**Dataset**	**Architecture (layers)**	**Performance outcomes**
Pharmacology	Drug–drug interaction	ACDGNN (Yu et al., 2023)	Intermediate	Gene, disease, pathway KG	HGNN with attention	AUC: 0.99–1.00; F1: 0.41–0.86; Acc: 0.67–0.92
Pharmacology	Drug repurposing	AD drug repurposing (Lin et al.)	Early	STRING, GO, CTD	GraphSAGE	AUC: 0.95–0.99; F1: 0.93–0.97; Acc: NR
Oncology	Glioma	VSA-GCNN (Pratap Joshi et al., 2025)	Intermediate	BraTS 2019/2020/2021	AlexNet, VSA, GCN with attention	AUC: NR; F1: 0.97–0.98; Acc: 0.92–1.00
Oncology	Breast cancer	MGNN (Gao et al., 2022)	Late	2,500 patients (gene expression, CNA, clinical data)	Temporal GNN on bipartite graphs; CCA fusion	AUC: 0.98; F1: NR; Acc: 0.95
Neuropsychiatry	Major depressive disorder	FC-HGNN (Gu et al., 2025)	Intermediate	ABIDE; REST-meta-MDD	GNN with attention	AUC: 0.95–1.00; F1: 0.93–1.00; Acc: 0.92–1.00
Neuropsychiatry	Alzheimer's disease	CsAGP (Tang C. et al., 2023)	Intermediate	ADNI1/GO; ADNI2	CNN; vision transformers; GNN with attention	AUC: 0.99–1.00; F1: NR; Acc: 0.94–0.99
Epidemiology	HAI transmission	MDRE-TransGraph (Gouareb et al., 2023)	Early	MIMIC-III	GNN with attention; GraphSAGE	AUC: 0.89–0.96; F1: NR; Acc: 0.84–0.97
Epidemiology	COVID-19 outcomes	Adaptable GCNN (Tariq et al., 2025)	Intermediate	EUH COVID-19 cohort	DenseNet-121; GraphSAGE; LSTM	AUC: 0.58–0.92; F1: NR; Acc: NR
Clinical	Comorbidity prediction	Multitask comorbidity GCN	Intermediate	Imaging + genomics + clinical notes	CNN; BERT; GNN	AUC: 0.96; F1: 0.93; Acc: 0.95
Clinical	Diabetic Retinopathy	DRdiag (Zedadra et al., 2025)	Intermediate	APTOS 2019; MESSIDOR	CNN; GNN	AUC: NR; F1: 0.96; Acc: 0.96–0.98
Genomics	ncRNA–miRNA interaction	ncRNAInter (Zhang et al., 2022)	Intermediate	lncRNASNP2; miRBase v22.1; GENCODE v38	GraphSAGE with neighbor sampling	AUC: 0.97–0.99; F1: 0.93–0.96; Acc: 0.93–0.96
Genomics	lncRNA–miRNA Interaction	GCNCRF (Wang et al., 2022)	Intermediate	lncRNASNP2; LncACTdb 3.0; LNCipedia; miRBase	CRF + GNN with attention	AUC: 0.88–0.95; F1: 0.13–0.14; Acc: 0.97–0.98

NR, Not reported by the original study.

In terms of datasets, population-level forecasting relies on datasets such as JHU CSSE, ILINet, OxCGRT, and social media signals (Qiu et al., 2024; Xie et al., 2023; Tran et al., 2024; Luo et al., 2025). Clinical prediction is often validated on MIMIC III and MIMIC IV for mortality, readmission, sepsis, and length of stay, and on institutional cohorts for triage and dynamic biomarker prediction (AL-Sabri et al., 2024; Tang S. et al., 2023; Ghanvatkar and Rajan, 2023; Christos Maroudis et al., 2025; Begum, 2024; Zhang et al., 2023). Imaging-heavy ophthalmology and retinal screening use APTOS and MESSIDOR and report gains when CNN features are integrated into patient similarity or knowledge graphs (Gao et al., 2024; Zedadra et al., 2025). Oncology combines TCIA archive and disease-specific collections for radiology, whole slide pathology, and multi-omic cohorts for survival modeling (Peng et al., 2024; Alzoubi et al., 2024; Yan et al., 2024; Gao et al., 2022). Gene regulatory and interaction studies rely on LncACTdb, LNCipedia, miRBase, ncRNASNP, HMDD, and GENCODE, which support sequence pretraining and heterogeneous graph construction (Wang Z. et al., 2023; Wang et al., 2022; Wang and Chen, 2023; Zhang et al., 2022).

The most prevalent layer types include GraphSAGE, GCN, GAT, and heterogeneous GNNs. Temporal encoders at the node level include LSTM, GRU, and temporal GNNs. Attention is used to weight neighbors and modalities. In epidemic forecasting, temporal encoders feed attention-based graph layers (Qiu et al., 2024; Xie et al., 2023; Tran et al., 2024; Luo et al., 2025). In clinical prediction, GraphSAGE and heterogeneous GNNs are combined with BiGRU or Transformer text encoders and time-aware designs (AL-Sabri et al., 2024; Tang S. et al., 2023; Begum, 2024; Boschi et al., 2024). In oncology, attention GNNs integrate imaging and omics (Alzoubi et al., 2024; Peng et al., 2024; Yan et al., 2024). Gene interaction models pair GraphSAGE with Doc2Vec k-mer embeddings, CRF layers, and multi-channel attention (Wang Z. et al., 2023; Wang et al., 2022; Wang and Chen, 2023; Zhang et al., 2022). Alzheimer's, COVID-19 Outcomes, and Drug-Target Prediction exhibit the highest layer type diversity, with 90%, 70%, and 60% of the models respectively combining multiple layer types, reflecting their complex multimodal requirements, as illustrated in the varied fusion strategies of Figure 3C. GNN + attention has the highest prevalence across included studies (63%), with CNN/Conv following closely with an incidence of 40% across studies, particularly in tasks like Alzheimer's and COVID-19 outcomes.

Forecasting tasks tend to model spatiotemporal data using intermediate fusion that aligns mobility and case signals with graph dynamics (Qiu et al., 2024; Xie et al., 2023; Tran et al., 2024; Luo et al., 2025). Operational and clinical tasks embed structured EHR, notes, images, and vitals with modality-specific encoders, which are fused in graph layers with attention (AL-Sabri et al., 2024; Tang S. et al., 2023; Valls et al., 2023; Ghanvatkar and Rajan, 2023; Zhang et al., 2023; Christos Maroudis et al., 2025). Neuropsychiatric tasks combine temporal encoders with imaging, electrophysiology, language, and omics within subject or population graphs with attention mechanisms (Cai et al., 2025; Liu et al., 2024; Li et al., 2024). Temporal encoders concentrate on time-dependent problems, including epidemic forecasting (75%) and COVID-19 outcomes (67%). A large overlap between attention mechanisms and temporal encoders has been observed in epidemic forecasting (75% attention; 75% temporal), ICU length of stay, ovarian cancer, prescription prediction, sepsis trajectory modeling, and neurodegenerative disease (Figures 2C, D, 3A).

Attention mechanisms and modality-specific encoders such as CNNs, RNNs, and graph layers that retain spatial, temporal, and relational structure correspond to higher predictive reliability across biomedical settings (Table 7). Attention-based intermediate fusion appears in most high-performing systems, particularly in tasks requiring integration of structured molecular features, clinical text, and imaging. Architectures combining GraphSAGE, GCN, or heterogeneous GNN layers with temporal or vision encoders achieve the strongest AUC and accuracy ranges in genomics, neuropsychiatry, and oncology. Domains with well-defined structural priors, such as ncRNA–miRNA prediction and drug–drug interaction modeling, show tighter performance bounds, whereas models operating on heterogeneous EHR or epidemiological data exhibit broader variability.

This review has several limitations. Marked heterogeneity in cohorts and nomenclature limits cross-study comparability and meta-analytic potential. Our harmonized taxonomy (early/intermediate/late fusion, layer families) may introduce classification error for mixed or sparsely described architectures, and many abstractions rely on self-reported methods without code or full graph-construction details. External validity is often weak, since numerous studies lack external validation. Widely used datasets (e.g., MIMIC, ADNI, ABIDE, and public KGs) may carry sampling biases that may hinder generalization. Finally, we did not apply a formal risk-of-bias tool or rerun models, as the main scope of this review is to build an understanding of how multimodal medical data is being integrated in GNNs.

## 9 Conclusion

GNNs offer a robust framework for modeling complex relationships across diverse modalities such as electronic health records, medical imaging, genomic profiles, and clinical notes. By synthesizing advancements in drug discovery, cancer detection, mental health diagnosis, epidemiology, clinical risk prediction, and gene expression analysis, this review has highlighted GNNs' ability to enhance clinical decision-making by leveraging graph-structured representations to capture intricate relationships among patients, diseases, drugs, imaging, text, and biological entities. The integration of GNNs with deep learning models, such as CNN, LSTM, RNN, dimensionality reduction, machine learning, and optimization algorithms, enhances their ability to process diverse data modalities. Multiple fusion strategies, such as early, intermediate, late, and hybrid, are employed to fuse multimodal data into a unified prediction framework. However, data heterogeneity across modalities, varying in structure and noise levels, complicates graph construction and fusion, while resource-intensive computations pose scalability issues. Interpretability and causality are essential for clinical adoption, with attention-based mechanisms offering partial solutions but requiring further development. Real-world use of multimodal GNNs also faces regulatory and operational barriers. Many models rely on complex graph-construction choices and stochastic training procedures that limit reproducibility across institutions, while the absence of standardized evaluation criteria complicates regulatory review. Deployment requires attention to data governance, privacy compliance, and integration with existing clinical workflows. Ensuring model generalizability across diverse datasets, addressing data availability, and complying with ethical, privacy, and security regulations are additional constraints that are yet to be fully addressed.

Several research directions follow from the patterns identified in this review. First, causal GNNs are needed to disentangle mechanistic relations from observational correlations in multimodal biomedical graphs, particularly for tasks such as treatment effect modeling, disease progression, and drug interaction inference. Second, privacy-preserving federated graph learning is essential for cross-institutional multimodal datasets. Third, the field lacks standardized explainability benchmarks for subgraph attribution, modality-specific contribution, and stability under perturbation, which would allow systematic comparison across fusion architectures. Lastly, future benchmarks should evaluate fusion strategies under controlled data heterogeneity to determine when early, late, or hybrid designs offer measurable advantages to ensure that multimodal GNNs are mechanistically informative, privacy-aligned, and reproducible at clinical scale.

## Glossary

ABIDE, Autism Brain Imaging Data Exchange; ADNI, Alzheimer's Disease Neuroimaging Initiative; ADHD-200, ADHD-200 Consortium neuroimaging dataset; ANIC, Australian National Intensive Care dataset (as cited); APTOS, Asia Pacific Tele-Ophthalmology Society diabetic retinopathy dataset; CBIS-DDSM, Curated Breast Imaging Subset of DDSM; CBHS, Commonwealth Bank Health Society (private insurer cohort); CCLE, Cancer Cell Line Encyclopedia; CDC ILI ILINet, CDC Influenza-Like Illness/Outpatient ILI Surveillance Network; CMMD, Chinese Mammography Database (as cited); CTD, Comparative Toxicogenomics Database; DAIC-WOZ, Distress Analysis Interview Corpus—Wizard of Oz; DAUH, Dong-A University Hospital (as cited); DAVIS, Kinase inhibitor binding benchmark (Davis et al.); DDSM, Digital Database for Screening Mammography; DISNET, Disease Networks knowledge base; EUH, Emory University Hospital; FAERS, FDA Adverse Event Reporting System; GENCODE, Comprehensive gene annotation resource; GEO, Gene Expression Omnibus; GDSC, Genomics of Drug Sensitivity in Cancer; HCP, Human Connectome Project; HMDD, Human microRNA Disease Database; iCTCF, International COVID-19 CT dataset (as cited); IQ-OTHNCCD, IQ-OTH/NCCD lung cancer imaging datasets; JHU CSSE, Johns Hopkins University CSSE COVID-19 repository; KEGG, Kyoto Encyclopedia of Genes and Genomes; KIBA, Kinase Inhibitor BioActivity benchmark; KRI, Korea Research Institute COVID-19 cohort (as cited); LncACTdb, Long Non-coding RNA–Associated Competing Endogenous RNA Database; LNCipedia, Long Non-Coding RNA knowledge base; MESSIDOR-2, Retinal fundus dataset for DR screening; miRBase, microRNA sequence database; MIMIC–IIIMIMIC–IV, Medical Information Mart for Intensive Care v3 / v4; MODMA, Multimodal Depression Dataset; MSBB, Mount Sinai Brain Bank; ncRNASNP, Non-coding RNA Single Nucleotide Polymorphisms database; OASIS, Open Access Series of Imaging Studies; OCD (Ovarian), Ovarian Cancer Dataset; OxCGRT, Oxford COVID-19 Government Response Tracker; PDBP, Parkinson's Disease Biomarkers Program; PLCO, Prostate, Lung, Colorectal, and Ovarian Cancer Screening Trial; PPMI, Parkinson's Progression Markers Initiative; RepoDB, Drug repurposing database; REST-meta-MDD, REST-meta-MDD Consortium dataset; ROSMAP, Religious Orders Study and Memory and Aging Project; SEER, Surveillance, Epidemiology, and End Results (NCI); SPAIN-COVID, Spain COVID epidemiological dataset (as cited); STITCH, Search Tool for Interactions of Chemicals; STRING, Search Tool for the Retrieval of Interacting Genes/Proteins; Synthea, Synthetic patient EHR generator; TCIA, The Cancer Imaging Archive; TWOSIDES, Large drug–drug interaction side-effect dataset; WSI, Whole-Slide Images (pathology); DTI (imaging), Diffusion Tensor Imaging (distinct from Drug–Target Interaction); EEG, Electroencephalography; fMRI, sMRI, Functional, Structural Magnetic Resonance Imaging; PET, Positron Emission Tomography; WSI, WholeSlide Images (pathology); ADE, Adverse Drug Event; ASD, Autism Spectrum Disorder; DDI, Drug–Drug Interaction; DTI (task), Drug–Target Interaction (disambiguated from imaging DTI); DR, Diabetic Retinopathy; HAI, Healthcare-Associated Infection; ICU, Intensive Care Unit; LOS, Length of Stay; MDD, Major Depressive Disorder; BERT, Bidirectional Encoder Representations from Transformers; BioBERT, Biomedical BERT; BiGRU, Bidirectional Gated Recurrent Unit; BiLSTM, Bidirectional Long Short-Term Memory; CAL, Content-Aware Layer (paper-specific); CNN, Convolutional Neural Network; CRF, Conditional Random Field; DNPGF, Dual-Nonlocal Pyramid Graph Filter (paper-specific); GAT, Graph Attention Network; GCN, Graph Convolutional Network; GCNN, Graph Convolutional Neural Network (generic); GGNN, Gated Graph Neural Network; GIN, GINConv, Graph Isomorphism Network/convolutional layer; GNN, Graph Neural Network; GNNRAI, GNN with Region-Aware Integration (paper-specific); GraphSAGE, Graph Sample and Aggregate; Graph Transformer, Transformer architecture on graphs; GTN, Graph Transformer Network (define in text; some papers vary); HGAT, Heterogeneous Graph Attention Network; HCNN-MAFN, Hypergraph CNN with Multimodal Attention Fusion Network (paper-specific); HGNN, Heterogeneous Graph Neural Network; HeteroGCN, Heterogeneous Graph Convolutional Network; ICA, Independent Component Analysis; KNN, k-Nearest Neighbors; LAYOUTLM, Document layout–aware Transformer; LINE, Large-scale Information Network Embedding; LLM, Large Language Model; LSTM, Long Short-Term Memory; MacBERT, Chinese BERT variant; MLP, Multi-Layer Perceptron; RGCN, Relational Graph Convolutional Network; RNN, Recurrent Neural Network; RPN, Region Proposal Network; SCL, Semantic Convolutional Layer (paper-specific); SDNE, Structural Deep Network Embedding; ST-GNN/STGCN, Spatio-Temporal GNN/Spatio-Temporal GCN; Transformer, Self-attention neural network; U-Net, U-shaped convolutional encoder–decoder; VAE, Variational Autoencoder; VGAE, Variational Graph Autoencoder; ViT, Vision Transformer; VSA, Variational Spatial Attention (paper-specific); Early, Intermediate, Late Fusion, Fusion timing categories used in this review; GAN, Generative Adversarial Network; GAT (attention), Graph attention mechanism/layer; KG, Knowledge Graph; KGE, Knowledge Graph Embedding; Q-Learning (RL), Reinforcement Learning Q-learning; RL, Reinforcement Learning; ROI, Region of Interest.
