# Supplementary material for: Multimodal graph neural networks in healthcare: a review of fusion strategies across biomedical domains
Source: Front Artif Intell. 2026 Jan 9;8:1716706. doi: 10.3389/frai.2025.1716706 (PMC12827511; doi:10.3389/frai.2025.1716706)
Supplement: Supplementary file 1 [file Data_Sheet_1.pdf]

**S1 Table. Full database search strings (PubMed, Google Scholar, arXiv; 2020–2025-08-31)**

| Source | Query |
|---|---|
| PubMed (Title/Abstract) | ( ("graph neural network"[tiab] OR GNN[tiab] OR "graph convolution"[tiab] OR GCN[tiab] OR GraphSAGE[tiab] OR GAT[tiab] OR "heterogeneous graph"[tiab] OR HGNN[tiab]) AND (multimodal[tiab] OR "multi-modal"[tiab] OR fusion[tiab] OR "early fusion"[tiab] OR "intermediate fusion"[tiab] OR "late fusion"[tiab]) AND (healthcare[tiab] OR clinical[tiab] OR medicine[tiab] OR patient[tiab] OR hospital[tiab] OR EHR[tiab] OR pharmacology[tiab] OR drug[tiab] OR oncology[tiab] OR cancer[tiab] OR neuro*[tiab] OR Alzheimer*[tiab] OR Parkinson*[tiab] OR depression[tiab] OR schizophrenia[tiab] OR autism[tiab] OR epidemiology[tiab] OR "COVID-19"[tiab] OR genomics[tiab] OR miRNA[tiab] OR lncRNA[tiab]) ) AND ("2020/01/01"[dp] : "2025/08/31"[dp]) |
| Optional module filter | AND (attention[tiab] OR "graph attention"[tiab] OR temporal[tiab] OR "time series"[tiab] OR LSTM[tiab] OR RNN[tiab]) |
| Google Scholar (custom date range 2020–2025) | ("graph neural network" OR GNN OR "graph convolution" OR GCN OR GraphSAGE OR GAT OR HGNN) AND (multimodal OR "multi-modal" OR fusion) AND (healthcare OR clinical OR medicine OR patient OR hospital OR EHR OR pharmacology OR drug OR oncology OR cancer OR Alzheimer OR Parkinson OR depression OR schizophrenia OR autism OR epidemiology OR "COVID-19" OR genomics OR miRNA OR lncRNA) |
| Google Scholar (modules/tasks) | ("graph neural network" OR GNN) AND (attention OR "graph attention" OR temporal OR "time series" OR LSTM OR RNN) AND (clinical OR EHR OR oncology OR neuro OR epidemiology OR genomics OR "drug-drug interaction" OR "drug target" OR "epidemic forecasting" OR "length of stay" OR readmission) |
| arXiv | ("graph neural network" OR GNN) AND (multimodal OR fusion) AND (healthcare OR clinical OR medical OR EHR OR oncology OR neuro OR "COVID-19" OR genomics) |
