# Supplementary material for: Multimodal graph neural networks in healthcare: a review of fusion strategies across biomedical domains
Source: Front Artif Intell. 2026 Jan 9;8:1716706. doi: 10.3389/frai.2025.1716706 (PMC12827511; doi:10.3389/frai.2025.1716706)
Supplement: Supplementary file 2 [file Data_Sheet_2.pdf]

S2 Table. Detailed eligibility criteria and screening rules.

Inclusion	Primary research applying a GNN to a health/biomedical task; combines ≥2 modalities except when data was processed through multiple layer types (CNN +GNN); reports or allows inference of fusion type (early/intermediate/late); architectural description sufficient to identify attention and/or temporal encoders.
Exclusion	Single-modality GNNs; non-health domains; reviews/editorials/tutorials; inaccessible full text.
Outcomes	Any predictive/diagnostic/prognostic task relevant to healthcare (no performance meta-analysis attempted).
