# Supplementary material for: Multimodal graph neural networks in healthcare: a review of fusion strategies across biomedical domains
Source: Front Artif Intell. 2026 Jan 9;8:1716706. doi: 10.3389/frai.2025.1716706 (PMC12827511; doi:10.3389/frai.2025.1716706)
Supplement: Supplementary file 3 [file Data_Sheet_3.pdf]

S3 Table. Source list, access dates, and filters by database.

Databases	PubMed; Google Scholar (primary); arXiv (preprint sweep)
Timeframe	2020-01-01 to [2025-08-31]
Hand-searching	Reference lists of included studies and venue-targeted checks (PLOS, Frontiers, JMIR, IEEE, MICCAI/MIDL, KDD/AAAI/IJCAI)
